# Does Methacrylation Affect the Cytocompatibility of Chitosan Scaffolds for Oral and Bone Tissue Regeneration? A Systematic Review

**DOI:** 10.1021/acsomega.6c03964

**Published:** 2026-07-06

**Authors:** Alana Pinto Caroso Souza, Anderson Gomes Forte, Fernanda Rafaela Ribeiro, Juan Vitor Costa Leite, Mario Alexandre Coelho Sinhoreti, Américo Bortolazzo Correr

**Affiliations:** Dental Materials Division, Department of Restorative Dentistry, Piracicaba Dental School, 245124 State University of Campinas (FOP/UNICAMP), Av. Limeira, 901, 13414-903 Piracicaba, São Paulo, Brazil

## Abstract

Objective: to evaluate the cytocompatibility of methacrylated chitosan (CSMA)-based biomaterials used for oral and bone tissue regeneration and whether the available evidence allows the biological effect of methacrylation to be isolated. Methods: This systematic review followed PRISMA guidelines and was registered on OSF. Searches were performed in PubMed, Embase, Scopus, and Web of Science up to August 2025. In vitro, ex vivo, and in vivo studies evaluating CSMA-based scaffolds, hydrogels, membranes, or bioinks and reporting quantitative cytocompatibility data were included. Due to methodological heterogeneity, a structured qualitative synthesis was performed. Results: Fourteen studies published between 2015 and 2025 were included. Most evaluated photo-cross-linkable hydrogels or scaffolds for bone, oral, or related tissue engineering applications. The reported degree of methacrylation ranged from 13.96% to 70%, although five studies did not report this parameter. Cell viability was generally maintained above 85–100% after 24 h, with some studies showing increased metabolic activity or proliferation over longer periods, reaching approximately 200% after 7–21 days. Favorable outcomes were often observed in hybrid systems containing GelMA, collagen, graphene, nanoparticles, peptides, or exosomes; however, formulation, cell type, assay method, and photopolymerization differences limited direct attribution of these effects to methacrylation alone. Conclusion: CSMA-based biomaterials generally showed favorable cytocompatibility. However, because many studies used hybrid formulations and lacked formulation-matched nonmethacrylated chitosan controls, the isolated biological effect of methacrylation could not be fully determined.

## Graphical abstract

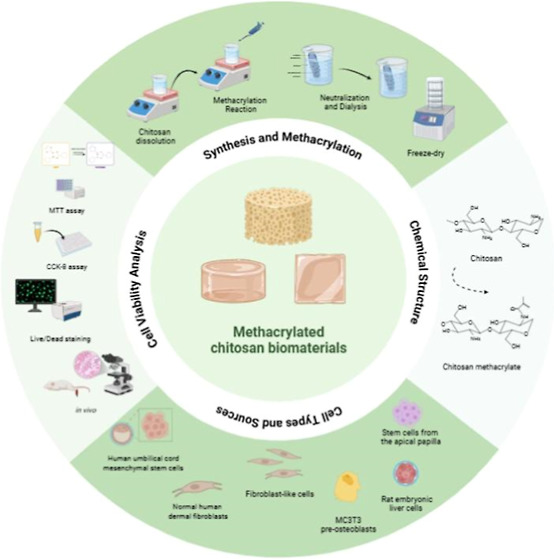

## 1 Introduction

Tissue engineering has become a central strategy in the regeneration of oral and bone tissues. Biopolymer-based scaffolds, hydrogels, and membranes are designed to reproduce the extracellular matrix environment, supporting cell adhesion, proliferation, and matrix deposition. Among natural polymers, chitosan stands out for its biocompatibility, biodegradability, and inherent antimicrobial properties, which make it particularly attractive for dentin–pulp complex regeneration, periodontal repair, and bone defect restoration.^1^

Nevertheless, chitosan exhibits limited mechanical integrity, uncontrolled degradation, and poor cross-linking reproducibility, restricting its long-term performance as a structural scaffold.^2^ To address these limitations, chemical functionalization through methacrylation has emerged as a versatile method. The incorporation of methacrylate moieties, typically via methacrylic anhydride or glycidyl methacrylate, permits light-induced cross-linking under mild conditions and allows precise modulation of network architecture and degradation kinetics.^3^ Such alterations inevitably influence key physicochemical parameters, including stiffness, hydrophilicity, and cross-linking density, which are known to affect cell adhesion, proliferation, and overall viability.^1,4^

Despite these advancements, the cytocompatibility of methacrylated chitosan remains an open question. Previous studies have reported divergent outcomes, moderate degrees of methacrylation (between 20–40%) have been associated with enhanced cell viability and spreading, whereas excessive cross-linking or high photoinitiator concentrations tend to reduce cell survival.^5,6^ Furthermore, the cellular response is strongly influenced by the biological context, as osteogenic cells generally tolerate stiffer matrices, while dental pulp and periodontal cells respond more favorably to softer and more compliant microenvironments.^7,8^

Although chitosan is widely recognized as a safe and bioactive material for regenerative applications, its methacrylation has not yet been systematically evaluated from a biological perspective. Cell viability is the primary determinant of biocompatibility, since materials with favorable mechanical or chemical properties may still fail clinically if they trigger cytotoxic responses or compromise cellular function.^9,10^ According to ISO 10993-5, in vitro cell viability assays represent the initial and most indicative stage in the biological evaluation of biomaterials, providing an essential screening of cytocompatibility prior to in vivo testing.^11^ Therefore, understanding how methacrylation modifies cellular behavior is crucial to ensuring the biological safety and translational potential of methacrylated chitosan scaffolds in regenerative dentistry. Even though several in vitro studies have evaluated cell viability in methacrylated chitosan systems, the available evidence remains fragmented and often qualitative, preventing the establishment of a clear consensus regarding their biological performance. Therefore, this systematic review aimed to provide a structured qualitative synthesis of the cytocompatibility of methacrylated chitosan-based scaffolds, hydrogels, membranes, and bioinks used in oral and bone tissue-engineering applications.

## 2 Materials and Methods

This systematic review was performed in accordance with the Preferred Reporting Items for Systematic Reviews and Meta-Analyses (PRISMA) guidelines.^12^ The study protocol was prospectively registered on the Open Science Framework (OSF) platform (https://osf.io/2wv4t/) under the DOI (10.17605/OSF.IO/XKDU6). The research question for this systematic review was developed using the acronym PICO (P) scaffolds, hydrogels, or membranes; (I) methacrylated chitosan scaffolds; (C) native chitosan, nonmethacrylated chitosan, blank controls, formulation-matched controls, or other CSMA-based formulations when direct native chitosan controls were unavailable; and (O) cell viability. The guiding research question for the development of this study was does methacrylation affect the cytocompatibility of chitosan scaffolds for oral and bone tissue regeneration?^13^

### 2.1 Eligibility Criteria

#### 2.1.1 Inclusion Criteria

Inclusion criteria comprised in vitro, ex vivo, and in vivo studies evaluating the cytocompatibility of methacrylated chitosan (CSMA)-based scaffolds, hydrogels, membranes, or bioinks intended for oral, bone, or related tissue engineering applications. Eligible studies were required to report quantitative cell viability, cytotoxicity, or cell proliferation data obtained using recognized biological assays, such as MTT, MTS, CCK-8, live/dead staining, Trypan Blue exclusion, or equivalent methods. Studies were included when CSMA-based materials were compared with native chitosan, nonmethacrylated chitosan, blank controls, formulation-matched controls, or other experimental CSMA-based formulations. Studies involving hybrid CSMA systems containing secondary bioactive components, such as GelMA, collagen, nanoparticles, peptides, exosomes, or other additives, were included only when the CSMA-containing formulation and the biological outcome could be clearly identified.

#### 2.1.2 Exclusion Criteria

Exclusion criteria included studies that did not use chitosan-based materials, did not report quantitative data, or had a lack of a control group. Editorials, letters to the Editor, theses, comprehensive reviews, and conference abstracts were also excluded.

### 2.2 Search Strategy

Structured and individualized search strategies were carried out in four electronic databases (PubMed/MEDLINE, Embase, Scopus, and the ISI Web of Science) up to August 2025. Additionally, a gray literature search was conducted using Google Scholar and OpenGrey. No restrictions regarding the language or year of publication were applied. A manual search was also performed by screening the reference lists and by identifying key authors or co-authors from the included studies. Experts in the field were consulted to ensure the comprehensiveness of the search. All database searches were performed by previously calibrated reviewers (A.P.C. and J.V.C.L.).

The following medical subject headings or text words were used: “scaffold”, “scaffolds”, “hydrogel”, “hydrogels”, “membrane”, “membranes”, “methacrylated chitosan”, “chitosan methacrylated”, “chitosan methacrylate”, “chitosan”, “methacrylic anhydride”, “cell viability”, “cytotoxicity”, “cytocompatibility”, “MTT”, “MTT assay”, “alamar blue assay”, “live/dead”, and “live/dead assay”. The advanced search tools were applied to combine the selected terms using Boolean operators (AND, OR), according to the specific format and rules of each database.

### 2.3 Study Selection

All references retrieved from the electronic databases were imported into the Rayyan platform (https://www.rayyan.ai), where duplicate records were automatically identified and removed. Two reviewers (F.R.R. and J.V.C.L.) independently screened titles and abstracts to exclude studies that did not meet the eligibility criteria, and any disagreements were resolved by a third reviewer (A.P.C.). Before the main screening phase, a pilot calibration was conducted using the first ten studies to ensure consistency in the selection process, yielding an inter-reviewer agreement of 90%.

### 2.4 Data Extraction

Three authors (A.G.F., F.R.R., and J.V.C.L.) independently extracted the most relevant methodological information from the included studies using a standardized data collection form. The extracted variables comprised: authors’ names, year of publication, and country. Methodological variables comprised study design, target tissue, type of material (scaffold, hydrogel, membrane, or bioink), material composition, methacrylation procedure, and degree of methacrylation (%). Biological variables included the cytotoxicity assay, evaluation period, cell line used, cytotoxicity outcomes, and main findings. When data were presented only in graphical form, the corresponding authors were contacted by email to obtain the numerical values. When numerical data were not available in the text, tables, or after contact with the corresponding authors, values were extracted from published graph images. This process was performed using PlotDigitizer. Graphs were calibrated according to the reported axis scales, and data points or bar heights were manually digitized. Figure-based data extraction was performed independently by two reviewers. Discrepancies greater than 5% were resolved by consensus or, when necessary, by consultation with a third reviewer. Because these values were obtained from digitized images rather than original raw data sets, they were considered approximate and identified as such in the data tables.

### 2.5 Quality Assessment

The risk of bias was independently assessed by two authors (A.G.F. and J.V.C.L.) using an adapted tool commonly applied in systematic reviews of in vitro studies, in conjunction with the Cochrane Collaboration’s guidelines 13–16. The assessment considered the following domains: presence of a control group, sample randomization, justification and reporting of sample size, consistency of experimental conditions across groups, adequacy and standardization of testing procedures and outcomes, blinding of the operator, appropriateness of the statistical analysis and outcome reporting, and completeness of reporting of key formulation parameters. This additional domain considered whether the degree of methacrylation, photoinitiator type and concentration, and photo-cross-linking conditions were adequately reported. Studies that did not report the degree of methacrylation were classified as high risk for this domain because this information is essential for interpreting dose–response relationships between methacrylation and cytocompatibility.

The risk of bias was categorized as “low” or “high,” and any disagreements were resolved by a third reviewer (A.P.C.). When sufficient details were provided for a specific parameter, it was classified as “low risk.” Conversely, when the information was missing or unclear, the parameter was rated as “high risk.” In cases where information was incomplete, the corresponding authors were contacted by email to clarify the specific parameter; if no response was received, the item was classified as “high risk.” Each study was then categorized according to the total number of parameters rated as “low risk,” as follows: 1–3 parameters, high risk; 4–6 parameters, medium risk; and 7–9 parameters, low risk.

Significant methodological heterogeneity was observed among the included studies. Therefore, meta-analysis was not performed because of differences in the cell type, assay method, evaluation time, scaffold composition, degree of methacrylation, photoinitiator system, and outcome reporting. A structured qualitative synthesis was conducted on the basis of the outcomes and data extracted from the studies that met the inclusion criteria. An important methodological consideration is that not all included studies directly compared CSMA-based materials with native or nonmethacrylated chitosan controls. Therefore, the synthesis was designed to evaluate the cytocompatibility profile of CSMA-based systems rather than to isolate methacrylation as the sole determinant of the cellular response. When hybrid formulations were evaluated, the potential contribution of secondary components, such as GelMA, collagen, nanoparticles, peptides, and exosomes, was considered during data interpretation.

## 3 Results

### 3.1 Selection of Sources of Evidence

A comprehensive search of PubMed, Embase, Scopus, and Web of Science, supplemented by gray literature sources (OpenGrey and Google Scholar), yielded 184 unique records after removing duplicates. During the screening of titles and abstracts, 137 studies were excluded for not fulfilling the predefined eligibility criteria, resulting in 47 articles selected for full-text review. After thorough evaluation, 33 articles were excluded because they did not focus on the dentin–pulp complex or bone regeneration. Additionally, one study was excluded as the full text could not be obtained despite repeated contact attempts with the corresponding authors. Ultimately, 14 studies satisfied all inclusion criteria and were incorporated into the qualitative synthesis of this review (Figure 1).

**Figure 1:**
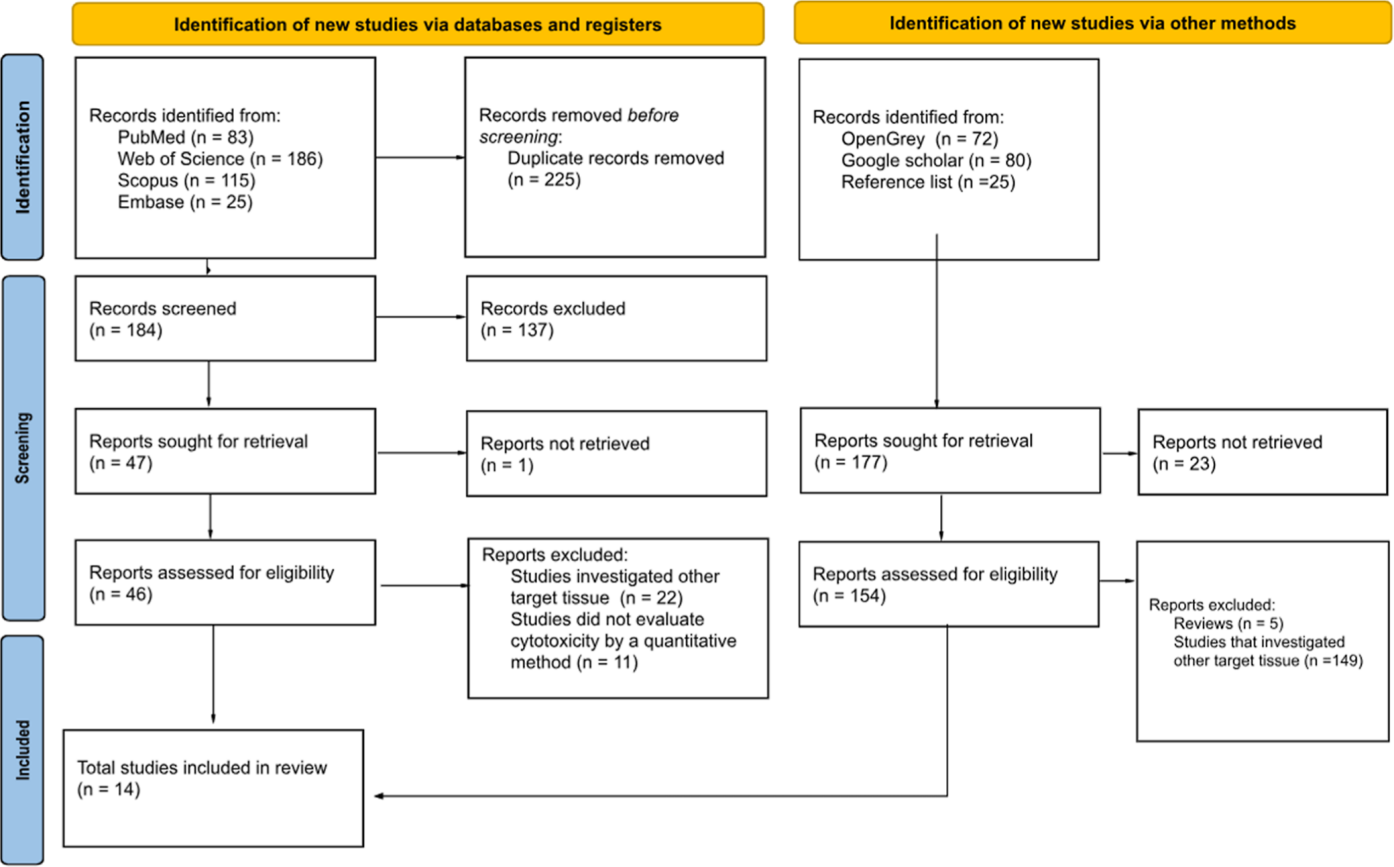
PRISMA 2020 flow diagram summarizing the identification and selection process.

### 3.2 Characteristics of Sources of Evidence

The publication years of the included studies ranged from 2015 to 2025 with most articles published in 2025. Among the 14 studies, all of them contained an in vitro component. Most investigations were exclusively in vitro, whereas three studies included in vivo analyses, and one included an ex vivo model. The studies reported different chemical routes to introduce methacrylate groups into chitosan, mainly through methacrylic anhydride, glycidyl methacrylate, nitro-based methacrylate derivatives, and NHS-activated methacrylate esters. Although these reagents differ in their reactive functional groups, their overall purpose is similar: to functionalize the hydroxyl and/or amino groups of chitosan, enabling subsequent light-induced cross-linking and the formation of stable three-dimensional hydrogel networks. The main methacrylation reagents, structural changes, and general fabrication workflows are summarized in Figure 2.

**Figure 2:**
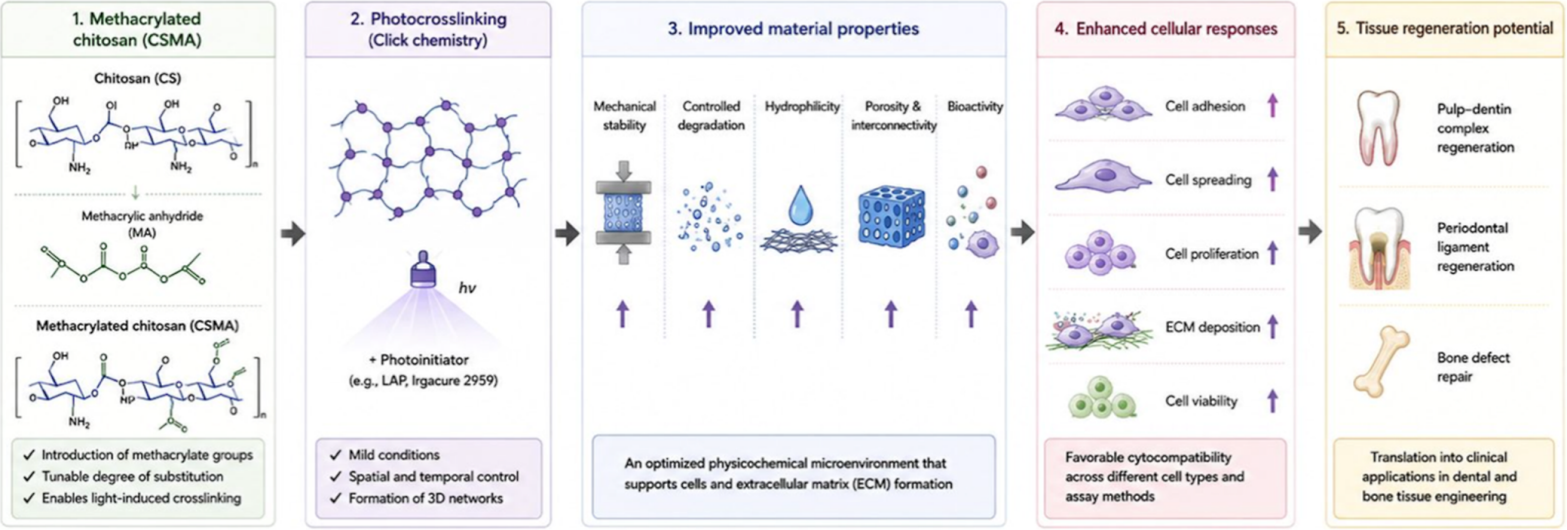
Schematic overview of methacrylated chitosan-based biomaterials. (1) Chitosan methacrylation; (2) photo-cross-linking; (3) improved material properties; (4) enhanced cellular responses; and (5) potential applications in dental and bone tissue regeneration.

The biomaterials were primarily applied within the contexts of tissue engineering,^17–22^ bone regeneration,^23–28^ and pulp–dentin complex regeneration.^29,30^ Biomaterials investigated included hydrogels (*n* = 11), scaffolds (*n* = 2), and membrane (*n* = 1) (Figure 3). Several studies evaluated hydrogels used as injectable bioinks for 3D printing of scaffolds^18,27–29^ whereas others referred to the printed construct simply as a scaffold, produced using a methacrylated chitosan bioink.^22,23^

**Figure 3:**
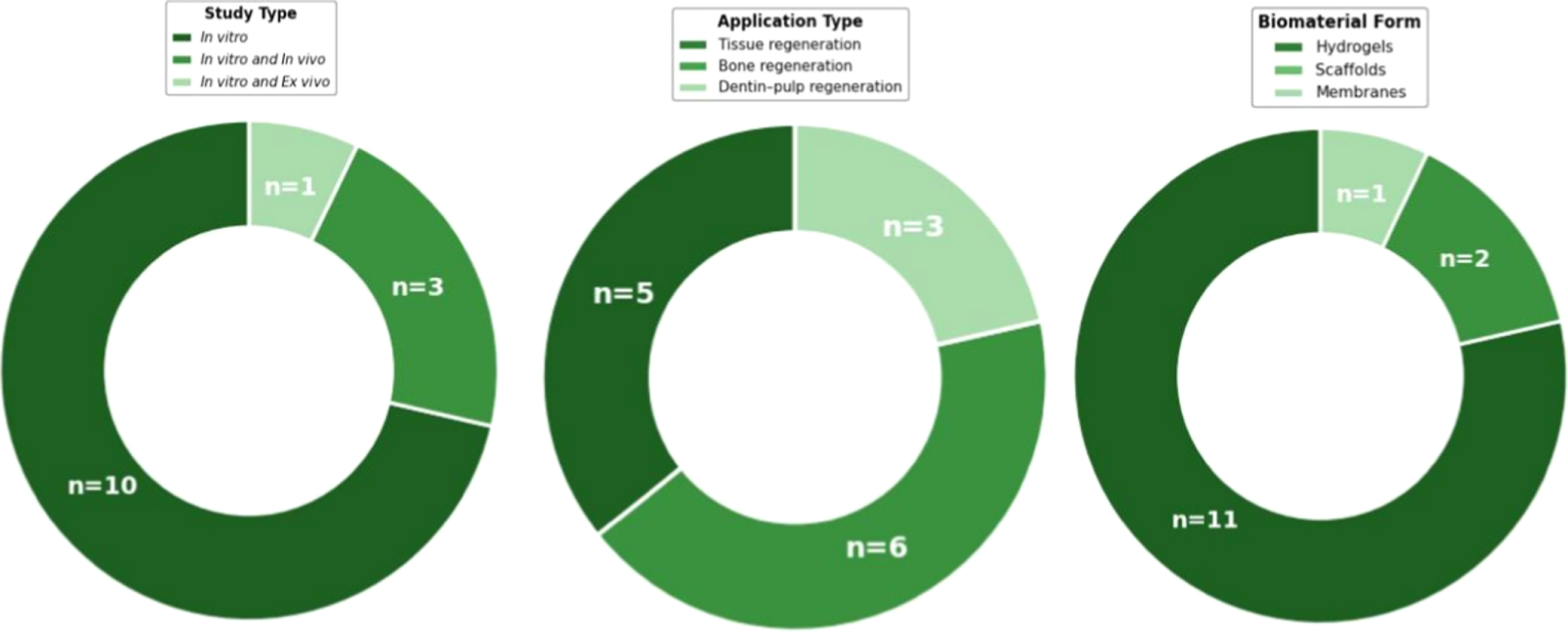
Distribution of study characteristics and biomaterial forms in methacrylated chitosan-based research.

A wide diversity was observed in the composition of the investigated biomaterials. Hydrogels were predominantly reinforced with natural polymers such as GelMA^24–26,28^ and collagen.^29,30^ Other formulations incorporated chemically converted graphene,^18^ gallic acid–grafted gelatin,^20^ hyaluronic acid,^21^ or maleilated chitosan.^17^ A unique approach was reported by Su et al. (2025), who developed a methacrylated chitosan matrix encapsulating exosomes derived from human umbilical cord mesenchymal stem cells (hUCMSC-Exos). Three-dimensional printed scaffolds were fabricated from composite systems, including chitosan reinforced with gelatin and laponite^22^ and methacrylated starch blended with glycerol.^23^ Only one study^19^ explored methacrylated chitosan in a membrane configuration, in which the polymer matrix was combined with jellyfish-derived collagen. The methacrylation approaches reported in the included studies varied according to the reagent used, reaction conditions, purification steps, and photo-cross-linking protocol. These strategies are summarized in Figure 4, which illustrates the main reagents, structural modification of chitosan, and general fabrication workflow leading to methacrylated chitosan-based hydrogels.

**Figure 4:**
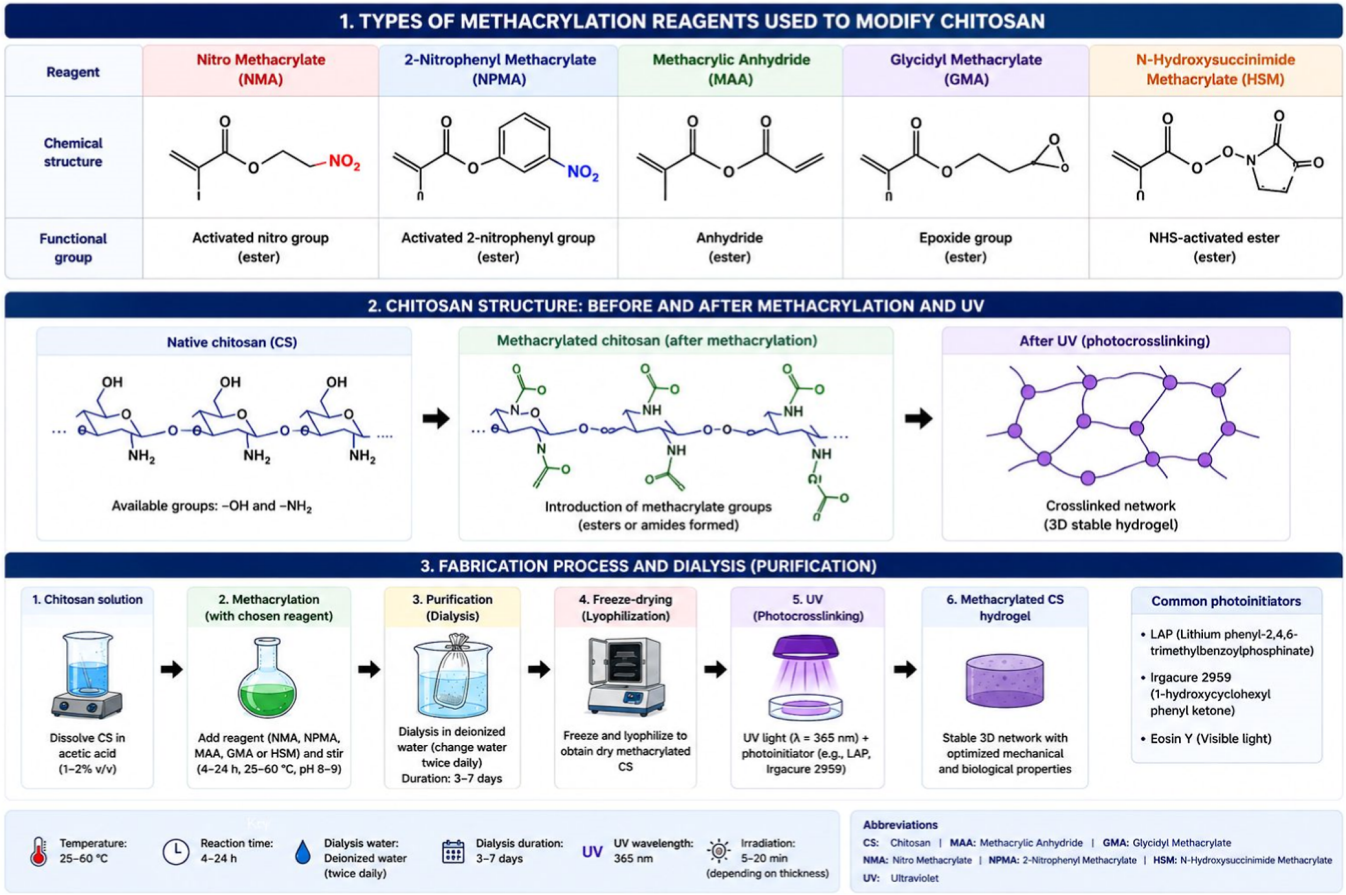
Overview of methacrylation reagents and the fabrication workflow for chitosan-based biomaterials: (1) the main reagents used for chitosan methacrylation; (2) the structural transition from native to methacrylated chitosan after UV-mediated photo-cross-linking; and (3) the general fabrication process, including chitosan dissolution, reagent addition, dialysis, freeze-drying, photo-cross-linking, and hydrogel formation.

The included studies used different cell types, including MC3T3 preosteoblasts,^22,24,28^ human umbilical cord mesenchymal stem cells (hUCMSCs),^21,27^ L929 fibroblast-like cells,^17,18,20,23^ rat embryonic liver cells 19, stem cells from the apical papilla (SCAP) 29,30, normal human dermal fibroblasts (NHDF) 25, and rat bone marrow-derived mesenchymal stem cells 26. Cytotoxicity assessment methods varied among the included studies and encompassed the MTT assay,^17,20,22,23,25,29,30^ the CCK-8 assay,^21,26,27^ Live/Dead staining,^18,20,26,28,30^ the Trypan Blue exclusion test 19, the Cell Growth Inhibition (CGI) assay,^18^ Actin/DAPI staining,^29^ the Calcein/PI Cell Viability and Cytotoxicity assay kit,^27^ and the CellTiter 96 MTS assay.^28^ Evaluation periods were predominantly 24 h,^17,20–27,29,30^ while some studies extended the observation to 3 days,^25,26,28^ 5 days,^14–18,22,24^ days 19, and up to 21 days.^27,29,30^

### 3.3 Summary of Results

The main characteristics of the included studies are summarized in Table 1. The reported degree of methacrylation varied considerably among studies, ranging from 13.96%^21^ to 26.08–53.85%,^20^ and approximately 70%.^18^ When expressed as molar ratios rather than percentages, values included 0.32,^24,29,30^ 0.27,^17^ 0.26,^26^ and 0.33.^25^ Several studies did not provide numerical data for the degree of methacrylation.^19,23,27,28^

**Table 1:** Main Methodological Data from Included Studies^a^

noo	type of study	context	biomaterial	material composition	methacrylation process	degree of methacrylation	cytotoxicity assay	evaluation period	cells	cytotoxicity outcomes (%)	main findings
Su et al., 2025	in vitro and in vivo	bone regeneration	hydrogel	TA–Ga network and CS-MA hydrogel with MSC-derived exosomes on titanium	chitosan (1.5 g) was dissolved in 4% acetic acid, reacted with 6 mL methacrylic anhydride for 12 h at 40 °C, dialyzed for 3 days, and freeze-dried to obtain CS-MA	not reported	CCK-8, calcein/PI cell, viability and cytotoxicity assay kit (Beyotime Biotechnology)	1, 4, 7, 14, and 21 d	umbilical cord mesenchymal stem cells (hUMSCs)	only immunofluorescence analysis figures are available	all coatings were noncytotoxic, with TA-Ga/CS-MA@Exo consistently enhancing cell viability/proliferation across RAW264.7, HUVEC, and MC3T3-E1, hemolysis <5%, and no in vivo tissue toxicity
Noohi et al., 2025	in vitro	dentin-pulp complex regeneration	hydrogel (applied as scaffold)	collagen-MA and chitosan-MA incorporating LLKKK18 or Tet213 peptides	chitosan (3% w/v) was dissolved in 2% acetic acid, reacted with methacrylic anhydride at a 1.2:1 molar ratio for 24 h, then dialyzed for 5 days and freeze-dried to obtain CS-MA	32%	MTT assay (Gibco) and actin/DAPI staining	1, 4, 7, 14, and 21 d	SCAP	cell viability >80% for ≤100 μM LLKKK18 and ≤0.1% Tet213; decreased to 54% and 71% at higher concentrations (200 μM, 0.2%); selected safe levels: 100 μM and 0.1%	AMP-loaded hydrogels maintained high SCAP viability with slight late-stage reduction linked to odontogenic differentiation; LLKKK18 enhanced differentiation, while Tet213 promoted cell migration, both showing excellent cytocompatibility
Gaihre et al., 2025	in vitro	bone regeneration	hydrogel (applied as scaffold)	Pi-OPF reinforced with acrylated montmorillonite (Ac-MMT) and a ChiMA-gelatin bioink	low molecular weight chitosan (1.5 g in 150 mL of 1% acetic acid) was reacted with 0.55 mL methacrylic anhydride, then dialyzed and lyophilized to obtain ChiMA	not reported	live/dead cell imaging kit (Thermo Fisher Scientific, MA) and quantitatively with CellTiter 96 MTS assay kit (Promega, WI)	0 h, 24, and 72 h	MC3T3 preosteoblasts	cell proliferation increased steadily from approximately 100% at 0 h to 200% at 24 h and around 260% at 72 h (% viability, extracted from graph image)	cell viability remained excellent after printing, with cells showing increased proliferation and a morphological shift from round to spindle-shaped over 72 h, indicating a favorable environment. MTS results confirmed progressively higher viability up to day 3
Zhou et al., 2025	in vitro	tissue engineering	scaffold	hybrid bioink of methacrylated starch with methacrylated chitosan +10%, 20%, 30%, and 40% of glycerol	methacrylated chitosan (CS-MA)—obtained by reacting chitosan (degree of deacetylation >90%) with methacrylic anhydride (MA) in 1% acetic acid solution	27%	MTT assay	24 h	L-929 cells	cell viability was 100% for the control, 87% for NCS-MA-CS, and 96% for NCS-MA-CS/10%Gly, indicating good cytocompatibility. (% viability, extracted from graph image)	the methacrylated starch–chitosan hybrid bioink demonstrated excellent cytocompatibility after 3D printing and photocross-linking, maintaining high cell viability. The incorporation of 10% glycerol further improved cell survival, bringing viability values close to those of the control group and confirming the material’s suitability for tissue engineering applications
Wang et al., 2025	in vitro and in vivo	wound regeneration	hydrogel	methacrylated chitosan (CSMA) with gallic-acid-grafted gelatin (GGA)	a 1% (w/v) chitosan solution (32 kDa) in 1% acetic acid was reacted with methacrylic anhydride (16 mL, 20% v/v) at 50 °C for 12 h. The product was dialyzed (14 kDa, 3 days), freeze-dried, and obtained as CSMA	43.13%	MTT and, live/dead assay	24 h	L929 cells	L929 cell viability after 24 h remained high (90–105%) for all gel formulations. (% viability, extracted from graph image)	the hydrogel demonstrated excellent cytocompatibility and bioactivity, showing no cytotoxic effects while supporting cell adhesion and proliferation. Its antibacterial and regenerative performance was remarkable, achieving complete bacterial eradication under NIR irradiation and promoting tissue regeneration through sustained Zn^2+^/Cu^2+^ ion release, which enhanced wound healing and overall biocompatibility
Deng et al., 2025	in vitro	wet tissue regeneration	hydrogel	methacrylated chitosan/hyaluronic acid coacervate	chitosan (3% w/v) was reacted with methacrylic anhydride (0.4 molar ratio) for 3 h at room temperature, followed by neutralization, ethanol washing, and freeze-drying to obtain CSMA	13.96%	CCK-8 assay	1, 4, and 7 d	human umbilical cord mesenchymal stem cells (hUCMSCs)	cell viability increased over time, reaching 100% on day 1, 120–140% on day 4, and 180–200% on day 7. (% viability extracted from graph image)	the CSMA/HA hydrogels exhibited excellent bioactivity and cytocompatibility, forming stable, adhesive, and mechanically robust networks without cytotoxic effects. Their strong tissue adhesion and wet stability demonstrate high potential for biomedical applications such as wound healing and tissue repair
Zertuche-Arias et al., 2025	in vitro and ex vivo	bone tissue regeneration	hydrogel	gelatin methacrylate and chitosan methacrylate	1.5% chitosan dissolved in 2% acetic acid reacted with methacrylic anhydride (1:6 molar ratio) for 24 h at 40 °C, followed by 4 days dialysis (12–14 kDa) and freeze-drying	32%	MTT assay and live/dead fluorescence in ex vivo calvaria model	1, 3, and 5 d	MC3T3-E1 preosteoblastic cells (in vitro); mouse calvarial bone explants (ex vivo)	cell viability increased over time, reaching approximately 90%, 88%, 87%, and 92% for GC, GCN, GCP, and GCNP on day 1; 93%, 95%, 94%, and 99% on day 3; and 96%, 98%, 97%, and 105% on day 5, respectively	all hydrogels were biocompatible, with GCNP (NAC + PAMP) showing the greatest cell viability, proliferation, and mineralization due to the combined effects of NAC on structure and PAMP on angiogenic and osteogenic activity
Morelli et al., 2024	in vitro	tissue engineering	membrane	chitosan methacrylate with jellyfish collagen	high molecular weight chitosan was methacrylated with methacrylic anhydride (4.6 mol ratio) at 40 °C for 12 h, followed by 5 days of dialysis and lyophilization to obtain purified CSMA	not reported	Trypan Blue exclusion test	7, 14, and 18 d	L929 cells	cell viability increased over time, reaching 100% for CSMA, 110–120% for CSMA/jCol30, and 120–130% for CSMA/jCol50 at day 7; 115–120%, 140–150%, and 155–165% at day 14; and 130–140%, 165–170%, and 180–190% at day 28, respectively	CSMA/jCol membranes were noncytotoxic and supported fibroblast viability and proliferation, with jCol addition (30% and 50%) markedly improving cell growth, adhesion, and organization compared to pure CSMA, indicating enhanced bioactivity and cytocompatibility
Noohi et al., 2022	in vitro	dentin-pulp complex regeneration	hydrogel	methacrylated chitosan and methacrylated collagen type I	chitosan (3% w/v in 2% acetic acid) was reacted with methacrylic anhydride (1.2 mol/mol repeating unit) for 24 h at room temperature, dialyzed for 5 days, and freeze-dried for 48 h	32%	MTT and live/dead assay	1, 4, 7, 14, and 21 d	SCAP	cell viability gradually increased, reaching 135% on day 1, 140% on day 4, 145% on day 7, and stabilizing at 150% by days 14 and 21	PRFe-loaded hydrogels significantly enhanced SCAP cell viability and proliferation (MTT assay, *p* < 0.05), maintaining a 1.4–1.5-fold increase over 21 days. Live/dead and F-actin/DAPI staining further confirmed improved cell adhesion and cytocompatibility
Zhang et al., 2019	in vitro and in vivo	bone regeneration	hydrogel	methacrylated chitosan bonded with polyhedral oligomeric silsesquioxane and methacrylated gelatin	chitosan (2 wt %) dissolved in 1% acetic acid, reacted with methacrylic anhydride (0.12 M) for 4 h at room temperature. Product dialyzed (3 days, water changes 3×/day) and lyophilized	26%	live/dead staining and CCK-8 assay	1, 2, and 3 d	rat bone marrow MSCs	cell viability remained high across all groups, with GelMA, NC, and DN showing 97%, 97%, and 98% on day 1; 97%, 98%, and 99% on day 2; and 98%, 98%, and 99% on day 3, respectively	live/dead staining revealed over 95% viable MSCs after 3 days in all hydrogels (Gel, NC, DN), demonstrating excellent cytocompatibility. The DN hydrogel showed significantly higher cell adhesion at 1–4 h (*p* < 0.05) and enhanced proliferation compared to gel and NC, confirming superior bioactivity and biocompatibility
Zhou et al., 2018	in vitro	tissue engineering	hydrogel (applied as scaffold)	maleilated chitosan/methacrylate poly(vinyl alcohol)	maleilated chitosan (MCS)—obtained by reacting chitosan (degree of deacetylation = 84.5%) with maleic anhydride in DMSO at 45 °C for 24 h, followed by neutralization and lyophilization	not reported	MTT assay	24 h	L-929 cells	relative cell viability (%) extracted from graph image: negative control: 100%; MCS/MPVA (3/1): 90%; MCS/MPVA (1/1): 99%; MCS/MPVA (1/3): 93%	all photo-cross-linked MCS/MPVA hydrogels showed high cell viability (>90%). The 1:1 ratio exhibited no significant difference from the control, while 3:1 and 1:3 showed slight but acceptable reductions. According to ISO 10993-5, all formulations are noncytotoxic and biocompatible for tissue engineering applications
Tugba Cebe et al., 2018	in vitro	bone regeneration	scaffold	methacrylated chitosan-laponite (MAC-Lp) and gelatin-laponite (MAG-Lp)	chitosan was methacrylated with methacrylic anhydride (0.5 mol ratio) under stirring for 3–4 h, followed by 7 days of dialysis and subsequent freeze-drying	not reported	MTS assay (Promega) for cell viability (24 h) and proliferation (1, 3, 7 days); fluorescence microscopy for adhesion	24 h, 3, and 7 d	MC3T3 preosteoblasts	cell viability and proliferation increased over time, with MAC and especially MAC-Lp showing the highest values at all points (*p* < 0.05–0.01), indicating superior bioactivity	all methacrylated scaffolds were biocompatible and noncytotoxic, supporting cell adhesion and proliferation. MAC, especially MAC-Lp, showed superior bioactivity, with cell viability reaching 200–220% compared to 100–120% for MAG-based scaffolds
Sayyar et al., 2017	in vitro	tissue engineering	hydrogel (applied as scaffold)	methacrylated chitosan associated with chemically converted graphene	methacrylated chitosan (ChiMA) was prepared by reacting chitosan with methacrylic anhydride at 60 °C, followed by 4 days of dialysis and freeze-drying	70%	live dead cell staining; CGI (assay)	48 h	L-929 cells	cell viability was 100% for the control, 95% for ChiMA, 98–100% for ChiMA +3 wt % CCG, and 0–10% for the positive control	cell viability and growth inhibition assays with L929 fibroblasts showed that ChMA and ChMA/CCG hydrogels were noncytotoxic and highly biocompatible. The addition of graphene (CCG) enhanced cell adhesion, confirming their suitability for tissue engineering applications
Saraiva et al., 2015	in vitro	bone and skin tissues	hydrogel	chitosan methacrylamide and gelatin methacrylamide	deacetylated chitosan (3% w/v) reacted with methacrylic anhydride (MA) for 4 h at room temperature, followed by 5 days of dialysis (12–14 kDa membrane) and freeze-drying	33%	MTS viability assay	24 and 72 h	NHDF	cell viability after 24 h was 85%, 83%, and 82% for ChMA/GelMA 2:1, 1:1, and 1:2, respectively, and after 72 h increased to 99%, 86%, and 90%, while K^–^ remained 100% and K^+^ 1%	ChMA/GelMA hydrogels were noncytotoxic and supported excellent NHDF adhesion, spreading, and proliferation. SEM and confocal imaging revealed dense, viable cell layers up to 100 μm depth, with the 1:2 composition showing slightly enhanced bioactivity due to higher gelatin content

a Not reported: studies that did not report the degree of methacrylation were classified as high risk in the risk-of-bias domain related to completeness of reporting of key formulation parameters.

The tested biomaterials exhibited high cell viability, typically above 85–100% at 24 h, with several studies reporting progressive increases in metabolic activity over time, confirming the cytocompatibility of methacrylated chitosan-based systems. In chitosan methacrylate (CSMA) and gelatin methacrylate (GelMA) hydrogels, cell viability remained comparable to controls at 24 h (95–100%).^18,20^ Some studies that included longer evaluation periods recorded viability rates exceeding baseline values, reaching 120–140% on day 4, 180–200% on day 7,^21^ and 110–190% between days 7 and 18 in CSMA/jCol composites.^19^

Double-network systems based on CSMA/POSS maintained stable viability around 97–99% throughout 3 days.^26^ Graphene- and gallate-modified matrices showed consistent values near 95–105% after 24 h.^18,20^ Three-dimensional printed or nanoparticle-reinforced scaffolds (MAC/MAG ± laponite) exhibited marked proliferation responses already at day 1 and maintained higher absorbance values at days 3–7, with statistically significant differences between groups.^22^

Polymer–plasticizer adjustments also preserved viability close to the control (87% for NCS-MA-CS and 96% when 10% glycerol was added) (Zhou et al., 2025). Studies involving peptide or bioactive-agent incorporation defined safe concentration ranges associated with >80% cell survival, while higher concentrations (200 μM or 0.2%) reduced viability to 54–71%.^29^ A few works reported only qualitative evidence (e.g., immunofluorescence micrographs or Live/Dead staining), which nonetheless indicated predominantly viable cells with preserved morphology and cytoskeletal integrity.^23,27^

Overall, the cytotoxicity profiles across studies consistently demonstrated that methacrylated chitosan-based biomaterials alone or in combination with GelMA, collagen, graphene, or bioactive nanoparticles were noncytotoxic and promoted cell adhesion, proliferation, and metabolic activity at different culture durations (24 h to 21 days). These findings were confirmed by multiple complementary assays, including MTT,^17,20,22,25,26,29,30^ CCK-8,^21,26,27^ and Live/Dead staining,^18,20,26,28,30^ which uniformly supported the excellent cytocompatibility of these systems.

### 3.4 Quality Assessment

The overall methodological quality of the included studies was considered to be satisfactory (Figure 5). Most domains were rated as having a low risk of bias, particularly those related to the presence of control groups, standardized testing procedures, and an appropriate statistical analysis. Nevertheless, methodological weaknesses were observed in sample randomization and operator blinding, which were frequently classified as high-risk or insufficiently described.

**Figure 5:**
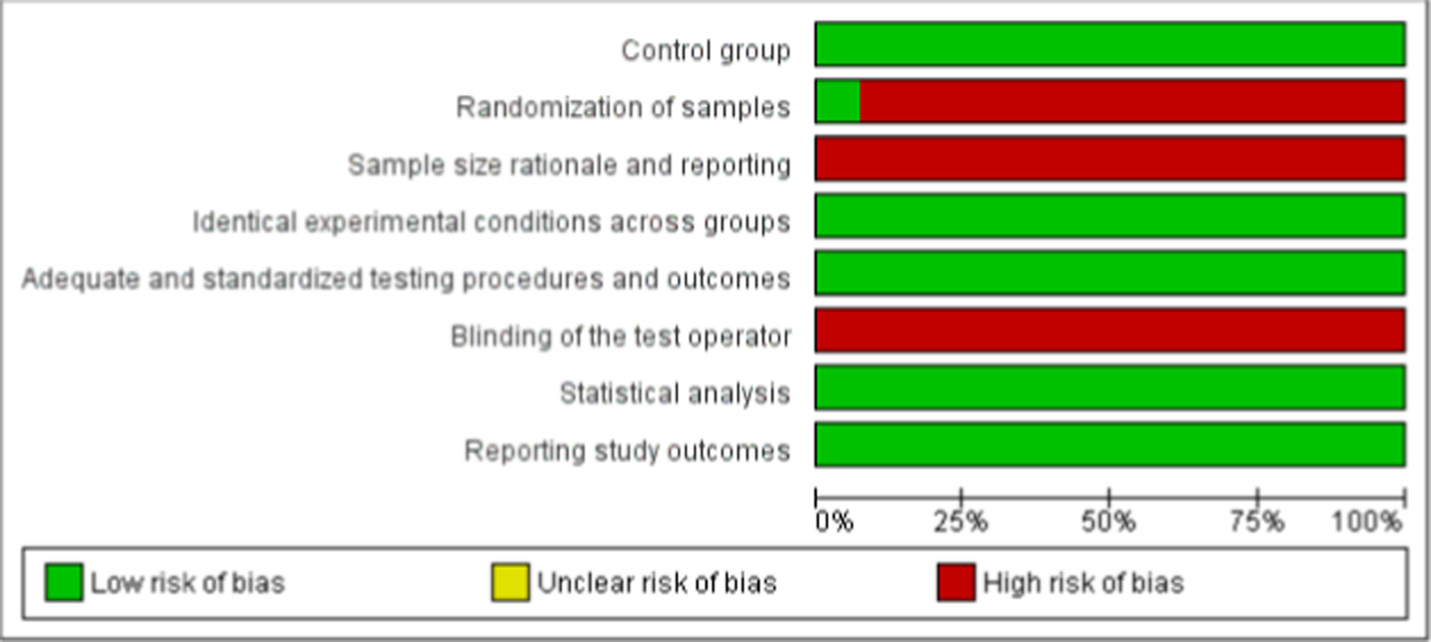
Overall distribution of risk of bias judgments for the included studies.

Substantial heterogeneity was observed among the included studies, particularly regarding the presence of control groups, uniformity of experimental conditions, standardization of testing procedures and outcome measures, and adequacy of statistical analyses and reporting. Meta-analysis was not performed because of differences in the cell type, assay method, evaluation time, scaffold composition, degree of methacrylation, photoinitiator system, and outcome reporting. Therefore, a structured qualitative synthesis was conducted on the basis of the outcomes and data extracted from the studies that met the inclusion criteria.

Five studies did not report the degree of methacrylation and were therefore classified as high risk in the domain related to completeness of reporting. This incomplete reporting limited cross-study comparison and prevented a robust dose–response interpretation of the relationship between methacrylation degree and cytocompatibility.

To explore this relationship, a descriptive dose–response map was constructed using studies that reported both the degree of methacrylation and percentage viability values (Figure 6). Overall, cell viability/metabolic activity remained favorable across a broad range of methacrylation degrees. However, no clear dose–response pattern could be established because of substantial heterogeneity in biomaterial composition, the assay method, cell type, and evaluation period. To avoid additional assumptions related to optical density normalization, Noohi et al. (2025) was not included in the descriptive dose–response map because cell viability was reported as optical density at 490 nm rather than as percentage viability. Nevertheless, this study was retained in the qualitative synthesis.

**Figure 6:**
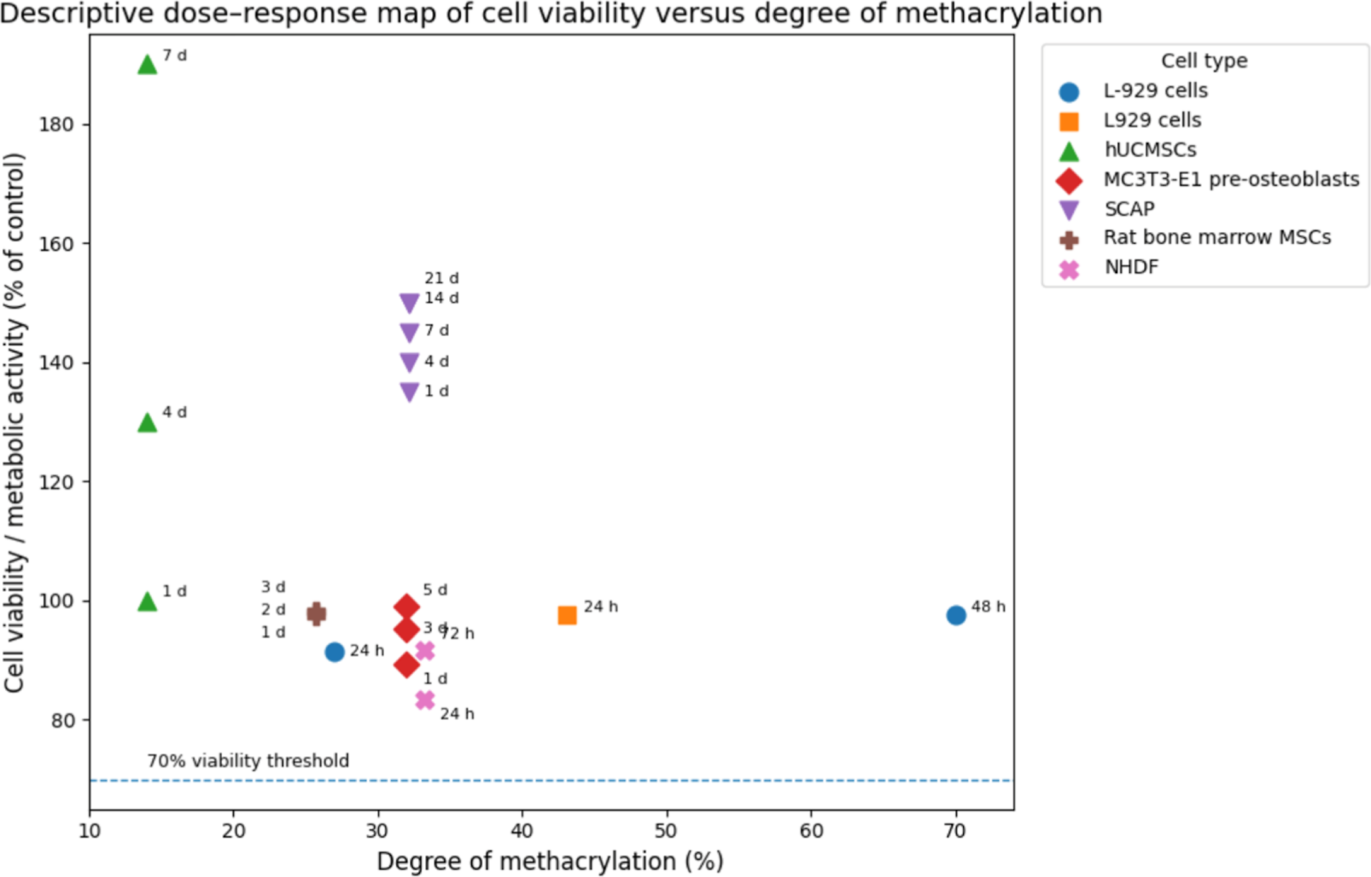
Descriptive dose–response map of cell viability/metabolic activity according to the degree of methacrylation. Note: Points represent reported or estimated percentage viability values from included studies, stratified by cell type and annotated according to evaluation time. When multiple formulations with the same degree of methacrylation were reported, mean or midpoint values were used for descriptive plotting purposes only. Studies without a reported degree of methacrylation or without directly available percentage viability values were not included in the plot.

## 4 Discussion

In this systematic review, 14 studies were analyzed to assess the cytocompatibility of methacrylated chitosan (CSMA)-based biomaterials for oral and bone tissue regeneration. Overall, the findings indicate that CSMA-based systems generally do not impair the cell viability. Several studies^19,21,22,24,25,28,29^ reported a progressive increase in metabolic activity and cell proliferation over time, supporting the favorable cytocompatibility of the CSMA-based biomaterials.

The temporal distribution of publications, spanning from 2015 to 2025, reveals a clear growth in CSMA-related research over the past decade, with a predominance of recent studies (2024–2025). This trend reflects technical advances in photo-cross-linkable biomaterials and biofabrication technologies, particularly 3D bioprinting and controlled photopolymerization. Early studies^18,22,25,26^ focused on basic cytotoxicity and physicochemical characterization, while more recent works^17,20,21,24,27,30^ explored hybrid and multifunctional formulations incorporating collagen, exosomes, or bioactive nanoparticles. This methodological evolution highlights the transition of CSMA from an experimental material to a promising biofunctional platform for bone, pulp, and soft tissue regeneration.

Most studies adopted in vitro designs (*n* = 11), three combined in vitro and in vivo approaches, and one included ex vivo analysis. CSMA was primarily investigated for bone and general tissue engineering,^17,18,20–22,24–28^ with fewer studies addressing dentin–pulp regeneration^29,30^ or soft tissue repair.^19^ This distribution demonstrates the versatility of CSMA as a matrix for mineralized and connective tissue reconstruction, owing to its tunable stiffness and degradation rate according to the target tissue.

The compositions varied widely, including hybrid CSMA–GelMA,^24–27^ collagen,^29,30^ graphene,^18^ hyaluronic acid,^21^ gallic acid-grafted gelatin,^20^ and maleilated chitosan.^26^ Photo-cross-linkable CSMA hydrogels exhibited the most consistent cytocompatibility profiles, likely owing to their uniform cross-linking density and efficient nutrient diffusion. Conversely, 3D-printed scaffolds and hybrid membranes were also biocompatible but showed greater variability, primarily influenced by differences in the polymer concentration, photoinitiator type, and light-curing efficiency. Overall, all formats maintained a high cell viability (>85%), confirming that the fabrication method modulates but does not compromise CSMA biocompatibility.

The methacrylation process varied among studies to tailor the desired properties of the final biomaterial. It was mostly carried out via the reaction of chitosan with methacrylic anhydride in acetic acid (1–4%) under moderate stirring at 25–60 °C, followed by dialysis (3–7 days) and lyophilization for purification.^20,24,25,27,30^ Methacrylic anhydride was the predominant reagent, while some studies used glycidyl methacrylate or premaleilated chitosan^26^ to modulate reactive site accessibility.

The degree of methacrylation ranged from 13.9%^21^ to 70%,^18^ with most studies reporting values between 25% and 45%.^17,20,24–26,29,30^ However, five of the included studies did not report this parameter, limiting cross-study comparison and dose–response interpretation. Studies reporting methacrylation degrees within the 25–45% range generally showed preserved cell morphology and high in vitro viability. Nevertheless, the descriptive dose–response map did not support the definition of an optimal methacrylation range. Although several studies within this range showed favorable cytocompatibility, comparable responses were also observed outside this interval. Therefore, the available evidence does not allow the identification of a unique optimal degree of methacrylation.

The analysis of the studies included in this review indicates that the photoinitiators used in the photo-cross-linking process of methacrylated chitosan matrices exert a direct influence on cell viability. In general, studies that explicitly reported the use of photoinitiators employed low-toxicity compounds such as Irgacure 2959 and LAP (lithium phenyl-2,4,6-trimethylbenzoylphosphinate), which are known for their excellent cytocompatibility when applied at concentrations below 0.1%.^3,5,6^ These photoinitiators provide high polymerization efficiency under UV or visible light and generate fewer reactive byproducts, thereby reducing the formation of reactive oxygen species and, consequently, minimizing cellular stress during the cross-linking process.^22^ In the studies,^20,21,24^ CSMA-based systems combined with GelMA, HA, or PAMP/NAC showed cell viability above 90% and a progressive increase in metabolic activity over time, reaching values close to 200% after 7 days. Similar results were observed,^17,25^ who reported cell viability ranging from 85% to 100% after photo-cross-linking, confirming that the use of cell-compatible photoinitiators and controlled light intensity are critical factors in preserving cellular integrity.

Cell viability assays varied but showed consistent outcomes. The MTT assay was most frequently used^17,20,23–25,29,30^ and MTS.^22,25^ These tetrazolium-based assays are endorsed by ISO 10993-5 as standard predictors of cytocompatibility. However, MTT may be less suitable for opaque hydrogels due to the formation of insoluble formazan crystals. In contrast, soluble-tetrazolium assays such as CCK-8 and MTS provide higher sensitivity and enable repeated, nondestructive measurements. Complementary qualitative assays—Live/Dead,^17,26,28^ Trypan Blue,^19^ and Calcein/PI,^27^ confirmed cell morphology and uniform adhesion. Given the physicochemical profile of CSMA, soluble, nondestructive assays like CCK-8 and MTS are the most suitable for long-term cytocompatibility studies. Although the included studies consistently indicated favorable cytocompatibility, the assay type should be considered when interpreting the magnitude of the reported outcomes. Tetrazolium-based assays, such as MTT, MTS, and CCK-8, primarily estimate metabolic activity and may therefore reflect changes in mitochondrial activity rather than cell number alone. In contrast, Live/Dead staining, Calcein/PI assays, and Trypan Blue exclusion provide complementary information on membrane integrity and cell distribution but are less suited for direct quantitative comparison across studies.

Despite methodological differences, results were consistent: cell viability remained above 85–100% at 24 h^18,20,25^ and increased over prolonged culture (7–21 days), reaching up to 180–200%.^19,21,29^ These findings indicate that CSMA not only maintains but also promotes cell proliferation and metabolism, likely due to its high hydrophilicity and efficient nutrient and oxygen diffusion.^2,9,10^

The cell types used reflected the target tissues: preosteoblastic MC3T3 cells^22,24,26,28^ and mesenchymal stem cells^21,27^ tolerated stiffer matrices, while SCAP^29,30^ and fibroblasts (L929 and NHDF)^20,23,25^ favored softer, hydrated environments. This correlation confirms the direct influence of cross-linking density and elastic modulus on cell adhesion and proliferation.^31,32^

In general, viability ranged from 85% to 100%, with higher values (95–105%) observed in composites containing GelMA, collagen, or graphene,^18,24,25,29^ regardless of the cell type or assay used. Collectively, these findings confirm that methacrylated chitosan is a cytocompatible and functional biomaterial capable of supporting cell adhesion, spreading, and proliferation without measurable cytotoxicity.^19–22,27^ Its favorable biological performance is attributed to the structural control provided by methacrylation, which modulates network cross-linking and stiffness while maintaining high hydrophilicity and permeability.^9,10^ Additionally, its ability to undergo photopolymerization under mild conditions and compatibility with 3D bioprinting technologies further enhance the translational potential of CSMA for personalized applications in mineralized and soft tissue engineering.^33^

Although cell viability was generally favorable across the included studies, cross-study comparison suggests that higher proliferation or metabolic activity was more often associated with composite formulations than with methacrylation alone. CSMA/MAC-based systems containing additional bioactive or extracellular matrix-derived components, such as peptides, exosomes, collagen, gelatin, jellyfish collagen, laponite, graphene, hyaluronic acid, or gallic-acid-grafted gelatin, tended to show enhanced adhesion, proliferation, mineralization, or tissue-related responses.^18,19,22,24,26–30^ In contrast, formulations without strong bioactive additives generally maintained viability within a noncytotoxic range, supporting the baseline cytocompatibility of CSMA/MAC-based platforms.^17,20,21,23,25^ Therefore, the available evidence indicates that CSMA/MAC is a cytocompatible photo-cross-linkable matrix, while enhanced biological performance likely depends on the combined effects of formulation composition, secondary bioactive components, scaffold architecture, degradation behavior, and photopolymerization conditions.

Most studies presented low risk of bias, with appropriate controls, standardized assays, and sound statistical analysis.^20,24,29^ The main limitations included lack of randomization and operator blinding, and insufficient reporting of methacrylation degree or photoinitiator concentration.^18,25,26^ Nonetheless, further in vivo and long-term studies correlating physicochemical properties with biological responses are still required to confirm the clinical safety and efficacy of these systems. In summary, current evidence indicates that methacrylation improves the structural stability and functional versatility of chitosan without compromising its biocompatibility, establishing CSMA as a promising biomaterial for bone, pulp, and periodontal regeneration.^19–21,29^

Although most included studies did not allow the biological effect of methacrylation to be isolated, three studies included MAC/CSMA-based control groups.^18,19,22^ Sayyar et al. compared ChiMA hydrogels with ChiMA/graphene hydrogels, Cebe et al. compared MAC scaffolds with MAC-laponite scaffolds, and Morelli et al. compared CSMA membranes with CSMA/jellyfish collagen membranes. These studies indicate that MAC/CSMA can serve as a cytocompatible base platform for the incorporation of secondary bioactive components. However, none of these studies included formulation-matched nonmethacrylated chitosan controls. Therefore, the specific biological effect of methacrylation was not fully isolated. Thus, the available evidence should be interpreted as supporting the cytocompatibility and versatility of MAC/CSMA-based composite platforms rather than demonstrating that methacrylation alone improves biological performance.

Additional physicochemical properties were extracted to support the mechanistic interpretation of cytocompatibility outcomes, including stiffness/elastic modulus, swelling behavior, cross-link density, hydrophilicity/contact angle, degradation kinetics, and compressive mechanical properties (Table S1, Supporting Information). To avoid conflating cytotoxicity with broader biocompatibility, differentiation markers, immunogenicity/inflammatory outcomes, genotoxicity end points, and residual methacrylate-related leachables were also extracted when reported (Table S2, Supporting Information). Only a limited number of studies assessed differentiation-related outcomes, mainly osteogenic or mineralization markers, such as ALP, ARS, calcium deposition, Runx2, OPN, OSX, and BSP. Immunogenicity-related outcomes were rarely reported and were generally limited to hemolysis, ex vivo tissue viability, histological inflammatory response, or an in vivo tissue response. No included study directly assessed genotoxicity end points, and none directly quantified residual methacrylate-related leachables. Therefore, the current evidence supports the cytocompatibility of CSMA/MAC-based systems but does not allow broad conclusions regarding complete biocompatibility, immunological safety, genotoxic safety, or residual monomer release.

An additional limitation is that not all included studies directly compared CSMA-based materials with native or nonmethacrylated chitosan controls. Thus, the isolated biological effect of methacrylation was not fully determined. Moreover, several formulations contained secondary components, such as GelMA, collagen, graphene, hyaluronic acid, peptides, exosomes, or bioactive nanoparticles, which may have contributed independently to the cell adhesion, proliferation, and metabolic activity. Therefore, the findings should be interpreted as evidence of the cytocompatibility of CSMA-based systems rather than as definitive evidence that methacrylation alone improves biological performance.

Based on the limitations identified in the included studies, future investigations should include native chitosan and formulation-matched nonmethacrylated controls to better isolate the biological effect of methacrylation. Authors should also report the degree of methacrylation, photoinitiator type and concentration, light exposure parameters, the polymer concentration, swelling behavior, stiffness, degradation kinetics, and residual methacrylate-related leachates. In addition, cytocompatibility assays should be complemented by differentiation markers, inflammatory/immunogenicity outcomes, genotoxicity end points, and long-term in vivo analyses. Such standardization would improve cross-study comparability and help define whether methacrylation itself, rather than secondary bioactive components or scaffold architecture, drives the biological response of CSMA/MAC-based systems.

## 5 Conclusion

The available evidence indicates that methacrylated chitosan-based biomaterials generally maintain favorable cytocompatibility across different cell types, assay methods, and culture periods. Most included studies reported cell viability above the cytotoxicity threshold, typically within or above the 85–100% range, with several studies showing preserved or increased metabolic activity over time. However, because many studies evaluated hybrid formulations and lacked formulation-matched nonmethacrylated chitosan controls, the isolated biological effect of methacrylation could not be fully determined. Therefore, the current evidence supports CSMA/MAC as a cytocompatible and versatile photo-cross-linkable platform for regenerative biomaterials, rather than demonstrating that methacrylation alone improves biological performance. Future studies should include native or nonmethacrylated chitosan controls, report the degree of methacrylation and photopolymerization parameters, and assess residual methacrylate-related leachables, immunogenicity, genotoxicity, and long-term in vivo safety.

## Supplementary Material



## Notes

Table summarizing the physicochemical properties of methacrylated chitosan-based biomaterials reported in the included studies and table summarizing extracted differentiation markers, inflammatory/immunogenicity outcomes, and residual methacrylate leachables reported in the included studies (PDF)
