# Supplementary material for: Does Methacrylation Affect the Cytocompatibility of Chitosan Scaffolds for Oral and Bone Tissue Regeneration? A Systematic Review
Source: ACS Omega. 2026 Jul 6;11(28):41041–53. doi: 10.1021/acsomega.6c03964 (PMC13392902; doi:10.1021/acsomega.6c03964)
Supplement: Supplementary file 1 [file ao6c03964_si_001.pdf]

Supporting Information *for* ACS Omega

Does methacrylation affect the cytocompatibility of chitosan scaffolds for oral and bone tissue regeneration? A systematic review

Alana Caroso-Souza*, Anderson Forte, Fernanda Ribeiro, Juan Vitor Leite, Mário Sinhoreti, and Américo Bortolazzo Correr

Dental Materials Division, Department of Restorative Dentistry, Piracicaba Dental School, State University of Campinas (FOP/UNICAMP), Av. Limeira, 901, 13414-903, Piracicaba, São Paulo, Brazil

---

AUTHOR INFORMATION

Corresponding Author

Alana Caroso-Souza (Alana Pinto Caroso Souza), DDS, MSc, PhD. Email: alanacaroso@gmail.com. ORCID: 0000-0002-2743-3012.

Authors

Anderson Forte (Anderson Gomes Forte), DDS. Email: andersongforte.ag@gmail.com. ORCID: 0009-0009-3378-3754

Fernanda Ribeiro (Fernanda Rafaela Ribeiro), DDS. Email: f290969@dac.unicamp.br. ORCID: 0009-0001-4963-662X

Juan Vitor Leite (Juan Vitor Costa Leite), DDS. Email: juanvitorleite@gmail.com. ORCID: 0000-0002-6069-6703

Mário Sinhoreti (Mário Alexandre Coelho Sinhoreti), DDS, MSc, PhD. Email: sinhoreti@fop.unicamp.br. ORCID: 0000-0002-1932-2902

Américo Bortolazzo Correr, DDS, MSc, PhD. Email: acorrer@unicamp.br. ORCID: 0000-0002-3306-7055.

Affiliation for all authors: Dental Materials Division, Department of Restorative Dentistry, Piracicaba Dental School, State University of Campinas (FOP/UNICAMP), Piracicaba, São Paulo, Brazil.

Manuscript ID: ao-2026-03964f.R1

Supplementary Table 1. Physicochemical properties of methacrylated chitosan-based biomaterials reported in the included studies.

Author and year	Stiffness / elastic modulus	Swelling behavior	Crosslink density	Hydrophilicity / contact angle	Degradation kinetics	Compressive mechanical properties
Su et al., 2025	NR	NR	NR	28.5–72.7°. All coatings were hydrophilic, with contact angles <90°.	NR	NR
Noohi et al., 2025	Young's modulus: 16.52 ± 0.97 to 18.51 ± 1.48 kPa. AMP incorporation had no significant effect.	Slight shrinkage; equilibrium after 10 h. Swelling at 24 h: 77.38 ± 3.76% to 87.16 ± 4.93%.	NR	NR	Evaluated for 6 days in lysozyme, collagenase, and dual-enzyme solutions. AMP-loaded hydrogels showed slower degradation in lysozyme.	NR
Gaihre et al., 2025	Compressive modulus increased with Ac-MMT: 50 to 150 kPa.	Printed lattice dimensions increased by 50% in cell culture medium.	NQ	NR	NR	Compressive strength at 70% strain increased from 0.06 to 0.35 MPa with Ac-MMT incorporation.
Zhou et al., 2025	NR	NR	NR	38.01 ± 1.47° to 50.22 ± 12.80°, indicating hydrophilic surfaces.	86–100% degradation after 48 h in α-amylase/lysozyme solution.	Compressive strength: 150–200 kPa; strain: 50–60%; toughness: 30–50 kPa.

Wang et al., 2025	NR	Equilibrium after 3 h; swelling ratio: 32–35%.	NR	NR	NR	NR
Deng et al., 2025	NR	Equilibrium within 10 h in PBS; lower swelling was associated with improved wet adhesion.	NR	NR	NR	Best performance at pH 5: compressive stress = 745 ± 34 kPa; compressive modulus = 2953 ± 90 kPa.
Zertuche-Arias et al., 2025	Young's modulus: GCNP = 55.26 ± 5.79 kPa.	GCNP showed the highest swelling ratio.	NQ	NR	GCNP showed the fastest degradation; GC retained >75% mass after 21 days.	Compressive modulus: GCNP = 55.26 ± 5.79 kPa; compressive strength: GCN = 151.79 ± 77.61 kPa; GCNP = 44.31 kPa.
Morelli et al., 2024	NR	NR	NR	CSMA: 84.4 ± 3.4°; CSMA/jCol30: 76.6 ± 2.6°; CSMA/jCol50: 71.2 ± 6.0°. Jellyfish collagen increased hydrophilicity.	Dissolution after 14 days: 52–61%; complete dissolution after 103 days.	NR
Noohi et al., 2022	Young's modulus: 14.03 ± 1.69 kPa at 20 s and 21.73 ± 3.24 kPa at 40 s for the 50/50 hydrogel.	Swelling decreased with longer light exposure and higher ChitMA/ColMA ratio.	NQ	NR	Evaluated for up to 6 days in lysozyme, collagenase, and dual-enzyme solutions.	Young's modulus: 5.24 ± 1.13 to 20.66 ± 2.27 kPa, depending on ChitMA/ColMA ratio.

Zhang et al., 2019	Compression modulus: DN = 108 kPa.	Equilibrium within 3 h; DN showed improved water uptake.	NQ	NR	Gel disappeared within 6 weeks; NC remained intact after 8 weeks; DN showed two-step degradation.	Compression stress: DN = 2.0 MPa at 89% strain; NC = 0.3 MPa at 78% strain.
Zhou et al., 2018	Storage modulus increased from 380 Pa to 8000 Pa with higher MPVA content.	Equilibrium within 50 min; water uptake: 6.65 ± 1.07 to 12.16 ± 0.46 g/g.	Indirectly assessed; sol fraction decreased from 0.170 ± 0.030 to 0.124 ± 0.009 g/g.	NR	NR	Compressive strength increased from 0.037 ± 0.012 to 0.169 ± 0.011 MPa with higher MPVA content.
Tugba Cebe et al., 2018	Elastic modulus NR; G' > G". Laponite increased rheological moduli.	Evaluated after 24 h in DPBS; MAC retained more water than MAG, while laponite reduced swelling.	NR	NR	Dissolution testing performed for 28 days, but no quantitative degradation rate was reported.	Compressive strength: MAG/MAC = 7–8 MPa; MAG-Lp/MAC-Lp = 14–15 MPa.
Sayyar et al., 2017	Young's modulus increased from 1.8 GPa for ChiMA to 4.6 GPa with 3 wt% CCG.	ChiMA swelling >260% after 48 h; CCG reduced swelling to ~160–205%.	NR	NR	NR	NR
Saraiva et al., 2015	NR	Swelling: 2:1 = 1823 ± 60%; 1:1 = 997 ± 49%; 1:2 = 889 ± 1%.	NR	2:1 = 62.4 ± 3.56°; 1:1 = 58.4 ± 2.21°; 1:2 = 64.9 ± 3.06°.	Weight loss after 21 days: 2:1 = 40%; 1:1 = 60%; 1:2 = 30%.	NR

Note. NR, not reported.

Supplementary Table 2. Extracted differentiation markers, inflammatory/immunogenicity outcomes, and residual methacrylate leachables reported in the included studies

Author and year	Differentiation markers	Immunogenicity / inflammatory response	Residual methacrylate leachables
Su et al., 2025	ALP staining/activity, OCN immunofluorescence, COL1a1 secretion, Alizarin Red staining, qPCR for ALP, OCN, COL1a1, and RUNX2, and Western blot for ALP, OCN, and COL1a1. TA-Ga/CS-MA@Exo enhanced osteogenic differentiation.	NR	NR
Noohi et al., 2025	AMP-loaded hydrogels promoted odontogenic differentiation of SCAP. Increased mineralized nodule deposition was observed for Gel-LLKKK18 and Gel-Tet213 compared with controls, along with upregulation of ALP, COL I, DSPP, DMP1, and VEGFA, and increased COL I and DSPP protein production.	NR	NR
Gaihre et al., 2025	ALP activity in MC3T3-E1 cells increased with Ac-MMT concentration, with the highest values for MMT-4% and MMT-6%, indicating enhanced osteoblastic differentiation.	NR	NR
Zhou et al., 2025	NR	NR	NR
Wang et al., 2025	NR	Inflammatory response was indirectly assessed by H&E staining. The treatment group showed reduced inflammatory infiltration and improved tissue regeneration. H&E staining of major organs showed no signs of inflammatory lesions or organ damage.	NR
Deng et al., 2025	NR	NR; hemocompatibility and hUCMSC cytocompatibility were assessed, but no specific inflammatory or immunogenic markers were reported.	NR

Zertuche-Arias et al., 2025	ALP and Alizarin Red staining were used. GCNP increased mineralized area and showed enhanced mineralization.	Ex vivo Live/Dead assay in calvarial explants was performed, but no specific cytokine or immunogenicity assay was reported.	NR
Morelli et al., 2024	Hepatic differentiation markers were evaluated, including albumin, CK18, and AFP. Glycogen storage and albumin synthesis were also assessed.	NR; inflammatory or immunogenic response was not directly evaluated.	NR
Noohi et al., 2022	NR; viability, proliferation, and cell morphology were reported, but differentiation markers were not confirmed.	NR	NR
Zhang et al., 2019	ALP, Alizarin Red staining, calcium content, BSP, Runx2, OPN, and OSX were assessed. Osteogenic proteins were evaluated by Western blot.	NR; in vivo bone regeneration was assessed, but no specific cytokine, inflammatory, or immune response assay was reported.	NR
Zhou et al., 2018	NR	NR	NR
Tugba Cebe et al., 2018	NR	NR	NR
Sayyar et al., 2017	NR	NR; biological assessment was limited to cytocompatibility, cell growth inhibition, viability, and L929 fibroblast adhesion/proliferation.	NR
Saraiva et al., 2015	NR; NHDF adhesion, proliferation, and cell internalization were evaluated.	NR; no direct inflammatory or immunogenicity assay was reported.	NR

Note. NR, not reported or not assessed. Residual methacrylate leachables were recorded only when residual methacrylate, methacrylic anhydride, or related extractables were directly quantified.
